# Supplementary material for: Role of Altered Metabolism of Triglyceride-Rich Lipoprotein Particles in the Development of Vascular Dysfunction in Systemic Lupus Erythematosus
Source: Biomolecules. 2023 Feb 21;13(3):401. doi: 10.3390/biom13030401 (PMC10046254; doi:10.3390/biom13030401)
Supplement: Supplementary file 1 [file biomolecules-13-00401-s001.zip › Supplementary table S1.pdf]

Supplementary Table S1. Clinical phenotypes and concomitant diseases of patients with systemic lupus erythematosus (SLE); n=51.

	n (%)
**Clinical phenotypes**	
Facial erythema	24 (47.1)
Polyarthritis	37 (72.5)
Alopecia	10 (19.6)
Pleuritis	11 (21.6)
Pericarditis	6 (11.8)
Myocarditis	1 (2)
Alveolitis	3 (5.9)
Raynaud syndrome	11 (21.6)
Nephritis syndrome	3 (5.9)
Antiphospholipid syndrome	3 (5.9)
Photosensitivity	10 (19.6)
Autoimmune haemolytic anaemia	5 (9.8)
Oral ulcers	2 (3.9)
Depression	1 (2)
Psychosis	0 (0)
Migraine	2 (3.9)
Lymphadenomegaly	6 (11.8)
Hepatomegaly	4 (7.8)
Splenomegaly	3 (5.9)
Myalgia	2 (3.9)
Polyneuropathy	2 (3.9)
**Concomitant diseases**	
Hypertension	2 (3.9)
Type 2 diabetes mellitus	1 (2)
Hypothyreosis	2 (3.9)
Mitral prolapse	2 (3.9)
Irritable bowel syndrome	1 (2)
