# Supplementary material for: Role of Altered Metabolism of Triglyceride-Rich Lipoprotein Particles in the Development of Vascular Dysfunction in Systemic Lupus Erythematosus
Source: Biomolecules. 2023 Feb 21;13(3):401. doi: 10.3390/biom13030401 (PMC10046254; doi:10.3390/biom13030401)
Supplement: Supplementary file 1 [file biomolecules-13-00401-s001.zip › Supplementary table S2.pdf]

Supplementary Table S2. Pearson correlations between laboratory parameters and augmentation index (Aix) in patients with systemic lupus erythematosus

**Variable**	**r**	**p**
Age (yrs)	0.27	0.08
Body mass index (kg/m^2)	0.23	0.44
PWV (m/s)	0.36	0.02
FMD (%)	-0.2	0.19
cIMT (cm)	-0.08	0.60
SS-A (U/mL)	0.01	0.92
SS-B (U/mL)	-0.01	0.95
C3 (g/L)	0.11	0.47
C4 (g/L)	-0.01	0.93
Interleukine-6 (mg/L)	0.07	0.60
C-reactive protein (mg/L)	-0.04	0.79
Total cholesterol (mmol/L)	0.30	0.054
LDL-C (mmol/L)	0.20	0.18
HDL-C (mmol/L)	-0.06	0.68
Triglyceride (mmol/L)	0.27	0.08
ApoA1 (g/L)	0.04	0.78
ApoB100 (g/L)	0.29	0.057
**Lipoprotein subfractions**		
VLDL (mmol/L)	0.31	0.04
IDL-A (mmol/L)	0.17	0.27
IDL-B (mmol/L)	0.29	0.05
IDL-C (mmol/L)	0.41	0.006
LDL1 (mmol/L)	0.29	0.059
LDL2 (mmol/L)	0.17	0.27
LDL3 (mmol/L)	0.12	0.42
Mean LDL size (nm)	-0.07	0.63
Large HDL (mmol/L)	0.01	0.93
Intermediate HDL (mmol/L)	-0.14	0.34
Small HDL (mmol/L)	0.03	0.87

Abbreviations: ApoA1: apolipoprotein A1; ApoB100: apolipoprotein B100; C3: complement 3; C4: complement 4; cIMT: carotid intima-media thickness; FMD: flow-mediated dilation; HDL: high-density lipoprotein; IDL: intermediate-density lipoprotein; IL-6: interleukine-6; LDL: low-density lipoprotein; PWV: pulse wave velocity; SS-A: anti–Sjögren's-syndrome-related antigen A autoantibodies; SS-B: anti–Sjögren's-syndrome-related antigen B autoantibodies; VLDL: very low-density lipoprotein.
